# Racial and Ethnic Differences in Trajectories of Hospitalization in US Men and Women With Heart Failure

**DOI:** 10.1161/JAHA.117.006290

**Published:** 2017-11-16

**Authors:** Matthew E. Dupre, Danan Gu, Hanzhang Xu, Janese Willis, Lesley H. Curtis, Eric D. Peterson

**Affiliations:** ^1^ Duke Clinical Research Institute Duke University Durham NC; ^2^ Department of Population Health Sciences Duke University Durham NC; ^3^ Department of Sociology Duke University Durham NC; ^4^ Population Division United Nations New York NY; ^5^ Duke School of Nursing Duke University Medical Center Durham NC; ^6^ Department of Community and Family Medicine Duke University Durham NC; ^7^ Division of Cardiology Department of Medicine Duke University Durham NC

**Keywords:** heart failure, hospitalization, race and ethnicity, Heart Failure, Race and Ethnicity

## Abstract

**Background:**

Prior studies have documented racial and ethnic disparities in hospitalization among patients with heart failure (HF). However, racial/ethnic differences in trajectories of hospitalization following the diagnosis of HF have not been well characterized. This study examined racial/ethnic differences in individual‐level trajectories of hospitalization in older adults with diagnosed HF.

**Methods and Results:**

Data from a nationally representative prospective cohort of US men and women aged 45 years and older were used to examine the number of hospitalizations reported every 24 months. Participants who were non‐Hispanic white, non‐Hispanic black, and Hispanic with a reported diagnosis of HF (n=3011) were followed from 1998 to 2014. Results showed a quadratic change in the number of reported hospitalizations following HF diagnosis, with an average of 2.36 (95% confidence interval [CI], 2.19–2.53; *P*<0.001) hospitalizations within 24 months that decreased by 0.35 (95% CI, −0.45 to −0.25; *P*<0.001) every 24 months and subsequently increased by 0.03 (95% CI, 0.02–0.05; *P*<0.001) thereafter. In men, there were no racial/ethnic differences in hospitalizations reported at the time of diagnosis; however, Hispanic men had significant declines in hospitalizations after diagnosis (Hispanic×time=−0.52; 95% CI, −0.99 to −0.05 [*P*=0.031]) followed by a sizeable increase in hospitalizations at later stages of disease (Hispanic×time^2^=0.06; 95% CI, 0.00–0.12 [*P*=0.047]). In women, hospitalizations were consistently high following their diagnosis and black women had significantly more hospitalizations throughout follow‐up than white women (black=0.28; 95% CI, 0.00–0.55 [*P*=0.048]). Racial/ethnic disparities varied by geography and the differences remained significant after adjusting for multiple sociodemographic, psychosocial, behavioral, and physiological factors.

**Conclusions:**

There were significant racial/ethnic differences in trajectories of hospitalization following the diagnosis of HF in US men and women. Racial/ethnic disparities varied by place of residence and the differences persisted after adjustment for multiple risk factors. The findings have important implications that may be crucial to planning the immediate and long‐term delivery of care in patients with HF to reduce potentially preventable hospitalizations.

**Clinical Perspective**

**What Is New?**

- There are significant racial and ethnic differences in trajectories of hospitalization following the diagnosis of heart failure in US men and women.
- Racial/ethnic differences in hospitalization vary by place of residence, and adjustment for multiple risk factors do not fully account for the differences.

**What Are the Clinical Implications?**

- Understanding trajectories of hospitalization has implications for planning the immediate and long‐term delivery of care in patients with heart failure.

Nearly 6 million Americans are currently diagnosed with heart failure (HF) and more than 1 million hospitalizations occur each year because of the disease.^1^, ^2^ According to recent estimates, HF is the leading cause of (re)hospitalization in older adults and has cost the United States an estimated $21 billion in direct annual medical costs.^1^, ^3^, ^4^ Racial and ethnic disparities in HF have been well documented^5^, ^6^, ^7^, ^8^ and a number of recent studies have shown that older black and Hispanic adults are hospitalized and readmitted more frequently for this condition than whites.^9^, ^10^, ^11^, ^12^, ^13^, ^14^ The reasons for these differences are largely unknown and racial/ethnic disparities largely persist after accounting for a variety of measures for the patients' background and clinical care.^15^, ^16^, ^17^, ^18^

Much of the existing literature on racial/ethnic differences in HF hospitalization has been based on hospital discharge records and administrative claims data from Medicare beneficiaries.^4^, ^10^, ^12^, ^13^, ^16^, ^17^, ^19^ Moreover, studies have not examined the number and timing of hospitalizations over the course of the disease, which may be crucial to planning the immediate and long‐term delivery of care in patients with HF. Therefore, research is urgently needed to advance our understanding of differences in patient‐level trajectories of hospital utilization to help catalyze new strategies of care and improve outcomes in the most vulnerable populations.

The purpose of this study is to provide the first prospective investigation of racial and ethnic differences in trajectories of hospitalization in US men and women with diagnosed HF. We used data from a nationally representative sample of older adults to examine individual‐level changes in hospitalizations reported over time among patients with recently diagnosed HF. We examined how trajectories differed among men and women and by race and ethnicity. We also examined whether the patterns of hospitalization changed after adjusting for numerous sociodemographic, psychosocial, behavioral, and physiological risk factors.

## Methods

### Study Population

Nationally representative data from the HRS (Health and Retirement Study) were used for analysis. The HRS is an ongoing prospective cohort study of US older adults sponsored by the National Institute on Aging and the Institute for Social Research at the University of Michigan.^20^, ^21^ The HRS was initiated in 1992 and has followed study participants biennially for more than 2 decades. Since 1998, the HRS has been supplemented with additional participants to add and replenish birth cohorts for a steady‐state design to maintain a representative (and aging) sample of US older adults. Details of the multistage sampling design, implementation, and response rates have been documented extensively elsewhere.^20^, ^21^ All HRS participants provided written informed consent and the study was approved by the University of Michigan Health Sciences Human Subjects Committee.

Data for the study were drawn from 36 289 HRS participants aged 45 years and older from 1998 to 2014. At each interview (every 24 months), participants were asked “Has a doctor told you that you have congestive heart failure?” This measure is the same as the one used for national estimates of diagnosed HF by the American Heart Association and the National Heart, Lung, and Blood Institute, among others.^1^, ^2^ The prevalence of HF in the HRS sample is also consistent with national rates of HF documented for this age group (Figure S1).^1^, ^22^, ^23^ The analyses were limited to respondents who reported an incident diagnosis during the 16‐year observation period (n=3603). We excluded 79 adults who identified their race/ethnicity other than white, black, or Hispanic and omitted an additional 380 adults who received a diagnosis before 1998. Thirty‐seven respondents were excluded because they lacked baseline data on hospitalizations and an additional 96 cases were dropped because of missing data on other baseline measures (Figure S2). The final analytic sample included 3011 older adults who contributed 8929 observations over the study period.

### Measurement

Multivariate models adjusted for several categories of previously identified risks as possible factors contributing to the associations. Sociodemographic characteristics included foreign‐born status, residence (urban or rural), geographic region (Northeast, Midwest, South, or West), educational attainment (<high school, high school graduate, or >high school), household income (in thousands), and health insurance coverage from any source (yes or no). Psychosocial and behavioral factors included marital status (married or not married), history of emotional or anxiety problems (yes or no), smoking history (never, current smoker, or former smoker), alcohol consumption (0, 1 or 2, or ≥3 drinks per day), vigorous physical exercise (<3 or ≥3 times per week), and does not take medication for diagnosed hypertension or other conditions (yes or no). Physiological factors included age at diagnosis, cardiovascular comorbidity (angina and acute myocardial infarction; yes or no for each), number of other chronic diseases (includes hypertension, diabetes mellitus, stroke/transient ischemic attack, lung disease, and cancer; range=0–5), body mass index (weight in kilograms divided by height in meters squared), and any limitations of activities of daily living (yes or no). The coding of study measures was facilitated by using HRS data files provided by RAND's Center for the Study of Aging and funded by the National Institute on Aging and the Social Security Administration.^21^

Preliminary analyses also included variables to adjust for study cohort, number of children, living alone, and health insurance type (eg, Medicare, Medicaid, private); however, results were not significant and the variables were dropped from the final models. Individual measures for disease comorbidity (eg, diabetes mellitus and hypertension) produced similar results in preliminary models; however, we used the summated comorbidity measure in the final analyses to avoid overfitting the models (because of high rates of disease in the HF sample) and for parsimony to maximize model fit. Alternative coding strategies were also assessed for continuous variables (eg, logged, polynomial, and grouped‐ordinal scales) and categorical variables (eg, different cut points, categories, and reference groups) and did not alter the central findings. Missing values were minimal at follow‐up measurements (0–1%) and all measures were time varying in the prospective analyses—with the exception of age at onset, sex, race/ethnicity, and foreign‐born status.

### Outcome

The primary outcome for analysis was the number of reported hospitalizations. At each survey wave (every 24 months), study participants were asked “How many different times were you a patient in a hospital overnight since the last interview?” Prior studies have shown that the self‐reported utilization of health care has substantial agreement with administrative claims data and medical records—with especially high concordance for reported hospitalizations (≥92% agreement in 1 year).^24^, ^25^, ^26^, ^27^, ^28^, ^29^, ^30^ Also consistent with previous studies, we included hospitalizations from all causes.^1^, ^4^, ^31^ Supplementary analyses were conducted to compare hospitalizations reported in the HRS with hospitalizations reported from national hospital discharge data, Medicare beneficiaries, and other US data (Tables S1 and S2). The results of these comparisons are consistent with the literature and suggest that overall hospitalization rates (per 1000 population) and the number of reported hospitalizations in the HRS are similar to other documented rates at these ages.^32^, ^33^, ^34^

All participants in the study were followed prospectively, and mortality was ascertained using the National Death Index and the HRS tracking file.^21^ Consistent with previous studies, the 5‐year mortality rate following HF diagnosis was ≈50% in HRS participants^1^, ^35^, ^36^ and whites had generally lower survival rates than nonwhites (Figure S3).^1^, ^2^, ^7^, ^10^

### Statistical Analysis

Baseline characteristics of the study participants were computed for men and women. Comparisons by race/ethnicity were calculated with chi‐square, ANOVA, and Kruskal–Wallis tests as appropriate. *P* values were based on 2‐tailed tests and considered statistically significant at *P*<0.05. Multilevel linear growth curve models (ie, mixed models) were then estimated to examine differences in trajectories of hospitalization following the diagnosis of HF.^37^, ^38^ We analyzed the longitudinal HRS data in a hierarchical framework to incorporate the individuals' repeated observations (level 1) nested within patients (level 2). Using mixed models with maximum likelihood estimation also has the advantage of accommodating the unbalanced data across time because it includes all participants regardless of their number of observations.^37^, ^38^, ^39^

First, we fit unconditional models with fixed and random linear (time) and quadratic (time^2^) functions that were added to the intercept‐only model (Table S3). Tests of model fit in preliminary analyses included higher‐order polynomials (eg, time^3^ and time^4^) and indicated that a quadratic function best parameterized the pattern of hospitalizations in the data. Race and ethnicity were then added to the models to account for between‐patient variations in hospitalizations—in mean levels (intercept) and changes (slope)—over time since diagnosis. The multilevel models were estimated separately for men and women to account for significant intercept and slope differences by sex, race, and ethnicity (Table S4). The first set of multilevel analyses estimated unadjusted differences in the number of hospitalizations in white, black, and Hispanic men and women. The second set of analyses estimated differences in hospitalizations while adjusting for sociodemographic, psychosocial, behavioral, and physiological factors. A final set of analyses included interactions to assess whether racial/ethnic differences in background characteristics (eg, place of residence and socioeconomic status) and/or other risk factors (eg, comorbidity and disability) contributed to differences in patterns of hospitalization following the diagnosis of HF in men and women.

Consistent with prior research, the multivariate models included a control variable for mortality in the estimation of the level 2 models.^40^, ^41^, ^42^ This approach has been shown to be effective in accounting for differences in the growth parameters (ie, hospitalizations over time) between patients who survived and those who died; it also controls for these differences on the effects of other covariates in the models.^40^, ^41^ A control for other attrition was also included in preliminary analyses and had no significant impact on the patterns of hospitalization. Finally, the data were not weighted because the study focused on a subsample of HRS respondents with diagnosed HF and the multivariate models included variables related to initial sample selection (age, sex, race, region) to produce unbiased estimates.^21^, ^43^ Analyses were conducted using Stata 14.2 (StataCorp LLC).

## Results

Baseline characteristics of the study participants are presented for men and women in Tables 1 and 2. In both sexes, whites were more likely to be native born, reside in nonurban areas, live outside of the south, and have greater socioeconomic resources than their nonwhite counterparts. There were few differences in health behaviors at baseline—with the exception of smoking—and psychosocial risks were generally greater in nonwhites than in whites. A diagnosis of HF in black and Hispanic men and women was received at significantly younger ages than in white men and women. Black and Hispanic participants also had more disease comorbidities, higher body mass index, and greater levels of disability than white participants with diagnosed HF. Black women had significantly more hospitalizations during follow‐up than white and Hispanic women (5.8 versus 4.7 hospitalizations, respectively); however, there were no racial/ethnic differences in the total number of hospitalizations in men.

**Table 1:** Baseline Characteristics of US Men With HF by Race and Ethnicity

	Total (n=1343)	Non‐Hispanic White (n=1047)	Non‐Hispanic Black (n=183)	Hispanic (n=113)	*P* Value
Sociodemographic background					
Foreign‐born	7.68	4.11	2.73	49.11	<0.001
Urban residence	68.26	64.37	81.42	83.04	<0.001
Geographic region					
Northeast	14.16	14.61	9.84	16.96	0.156
Midwest	26.60	29.99	20.77	4.46	<0.001
South	42.85	39.64	60.66	43.75	<0.001
West	16.39	15.76	8.74	34.82	<0.001
Level of education					
Less than high school	29.36	23.69	42.08	61.61	<0.001
High school graduate	32.86	34.38	34.43	16.07	
More than high school	37.78	41.93	23.50	22.32	
Household income, median (IQR)^a^	31.77 (38.71)	34.49 (38.14)	20.80 (36.00)	16.22 (23.51)	<0.001
No health insurance	3.43	2.77	4.37	8.04	0.011
Psychosocial and behavioral factors					
Not married	53.28	51.58	63.39	52.68	0.013
Emotional or anxiety problems	20.42	18.91	24.56	27.68	0.029
Smoking history					
Current smoker	12.67	10.79	20.77	16.96	<0.001
Former smoker	65.57	67.34	57.38	62.50	0.025
Alcohol consumption					
Never drinks	56.86	56.54	60.11	54.46	0.579
Drinks in excess	6.93	7.26	4.92	7.14	0.514
Inadequate exercise	88.60	88.35	89.62	89.29	0.858
Does not take medication(s)	6.78	6.88	6.01	7.14	0.900
Physiological factors					
Age at diagnosis, mean (SD), y	71.73 (11.01)	73.13 (10.54)	66.90 (11.04)	66.58 (11.57)	<0.001
Cardiovascular comorbidity					
Angina	47.24	46.51	45.36	57.14	0.087
Acute MI	50.52	49.95	45.36	64.29	0.005
Chronic illnesses, mean (SD), No.	1.74 (1.07)	1.70 (1.09)	1.89 (1.00)	1.86 (0.96)	<0.001
Body mass index, mean (SD)	28.01 (6.01)	27.80 (5.77)	28.59 (6.90)	29.02 (6.50)	<0.001
Any ADL limitations	38.00	36.29	40.44	50.00	0.014
Hospitalizations, mean (SD), No.^b^	4.64 (5.95)	4.66 (6.23)	4.74 (4.78)	4.23 (4.98)	0.838
Follow‐up time, median (IQR), y	4 (6)	4 (6)	4 (6)	6 (8)	0.422

Values are expressed as percentages unless otherwise indicated. ADL indicates activities of daily living; HF, heart failure; IQR, interquartile range; MI, myocardial infarction; SD, standard deviation.

a Reported in thousands of dollars.

b Includes the total number of hospitalizations reported during the study period.

**Table 2:** Baseline Characteristics of US Women With HF by Race and Ethnicity

	Total (n=1669)	Non‐Hispanic White (n=1195)	Non‐Hispanic Black (n=344)	Hispanic (n=130)	*P* Value
Sociodemographic background					
Foreign‐born	7.55	4.52	2.03	50.00	<0.001
Urban residence	69.62	64.27	80.81	89.23	<0.001
Geographic region					
Northeast	13.78	14.06	12.21	15.38	<0.001
Midwest	25.40	28.45	22.38	5.38	<0.001
South	46.14	42.68	60.17	40.77	<0.001
West	14.68	14.81	5.23	38.46	<0.001
Level of education					
Less than high school	37.75	32.97	43.02	67.69	<0.001
High school graduate	36.55	40.25	30.81	17.69	
More than high school	25.70	26.78	26.16	14.62	
Household income, median (IQR)^a^	17.37 (21.79)	19.68 (22.55)	12.39 (17.25)	11.29 (11.58)	<0.001
No health insurance	3.59	1.76	6.98	11.54	<0.001
Psychosocial and behavioral factors					
Not married	77.17	78.33	79.36	60.77	<0.001
Emotional or anxiety problems	30.14	29.46	29.65	37.69	0.148
Smoking history					
Current smoker	11.62	10.29	15.99	12.31	0.014
Former smoker	39.90	40.25	40.99	33.85	0.330
Alcohol consumption					
Never drinks	78.01	77.57	77.62	83.08	0.348
Drinks in excess	1.20	1.00	1.74	1.54	0.503
Inadequate exercise	94.01	93.56	94.48	96.92	0.283
Does not take medication(s)	6.17	6.53	5.23	5.38	0.630
Physiological factors					
Age at diagnosis, mean (SD), y	73.91 (12.00)	76.23 (11.21)	67.45 (11.51)	69.65 (12.86)	<0.001
Cardiovascular comorbidity					
Angina	45.00	44.52	47.38	43.08	0.578
Acute MI	36.79	36.74	35.17	41.54	0.439
Chronic illnesses, mean (SD), No.	1.86 (1.05)	1.82 (1.06)	1.99 (0.99)	1.87 (0.99)	<0.001
Body mass index, mean (SD)	28.57 (7.86)	27.42 (7.13)	32.12 (8.94)	29.77 (8.21)	<0.001
Any ADL limitations	51.59	48.45	56.1	68.46	<0.001
Hospitalizations, mean (SD), No.^b^	4.91 (6.10)	4.69 (5.84)	5.76 (7.18)	4.73 (5.12)	<0.001
Follow‐up time, median (IQR), y	4 (6)	4 (6)	6 (4)	4 (6)	0.074

Values are expressed as percentages unless otherwise indicated. ADL indicates activities of daily living; HF, heart failure; IQR, interquartile range; MI, myocardial infarction; SD, standard deviation.

a Reported in thousands of dollars.

b Includes the total number of hospitalizations reported during the study period.

**Figure 1:**
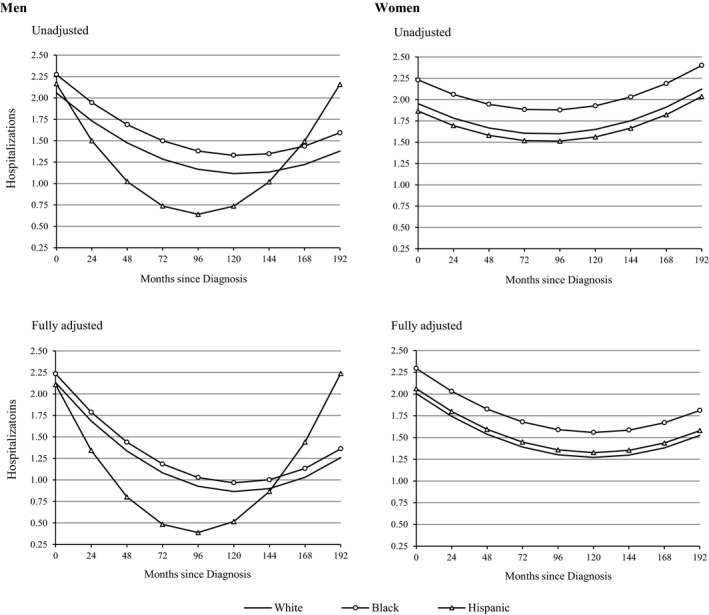
Predicted trajectories of hospitalization by race and ethnicity in US men and women with heart failure. Plotted from estimates shown in Table S3. Adjusted estimates account for foreign‐born status, geographic region, urban residence, education, household income, health insurance, marital status, anxiety/emotional problems, smoking history, alcohol consumption, exercise, medication use, age at diagnosis, cardiovascular comorbidity (angina and acute myocardial infarction), other comorbidities (hypertension, diabetes mellitus, stroke, lung disease, and cancer), body mass index, functional status, and mortality attrition.

Results from multilevel growth models showed that the number of hospitalizations exhibited quadratic change following HF diagnosis, with an average of 2.36 (confidence interval [CI], 2.19–2.53; *P*<0.001) hospitalizations at baseline that decreased by 0.35 (95% CI, −0.45 to −0.25; *P*<0.001) every 24 months and subsequently increased by 0.03 (95% CI, 0.02–0.05; *P*<0.001) thereafter (Table S3). Women had more hospitalizations than men and the trajectories of hospitalization differed significantly by sex and race/ethnicity. Figure 1 illustrates the individual‐level trajectories of hospitalization over time by race and ethnicity in men and women (plotted from estimates in Table S4). The unadjusted models (upper panels) show that the overall numbers of hospitalizations in men and women were elevated during the initial period after the diagnosis of HF. However, hospitalizations significantly declined during the subsequent period; followed by a significant increase in hospitalizations during latter stages of illness. In men, we found no racial/ethnic differences in hospitalizations at the time of diagnosis; however, we found that Hispanic men had significantly fewer hospitalizations than non‐Hispanic men during the major course of illness (Hispanic×time=−0.52; 95% CI, −0.99 to −0.05 [*P*=0.031] and Hispanic×time^2^=0.06; 95% CI, 0.00–0.12 [*P*=0.047]). Thereafter, the number of hospitalizations escalated in Hispanics during the latter stages of disease. In women, hospitalizations were consistently high following the time of their diagnosis. We also found that black women had significantly more hospitalizations than white and Hispanic women (black=0.28; 95% CI, 0.00–0.55 [*P*=0.048]), with differences that remained constant throughout the follow‐up period (ie, no change in slope). The fully adjusted models (lower panels) show that the racial and ethnic differences in hospitalizations remained largely unchanged after accounting for numerous covariates.

**Figure 2:**
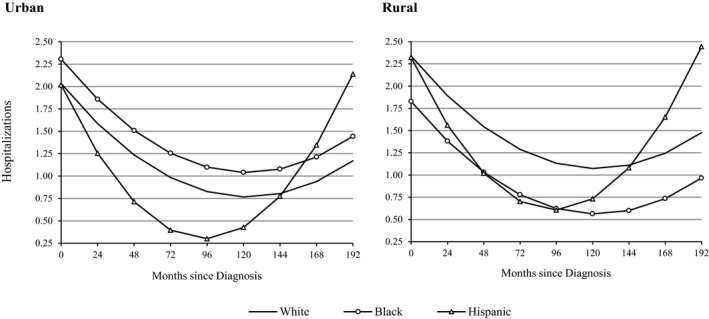
Predicted trajectories of hospitalization by race, ethnicity, and urban residence in US men with heart failure. Estimates account for foreign‐born status, geographic region, education, household income, health insurance, marital status, anxiety/emotional problems, smoking history, alcohol consumption, exercise, medication use, age at diagnosis, cardiovascular comorbidity (angina and acute myocardial infarction), other comorbidities (hypertension, diabetes mellitus, stroke, lung disease, and cancer), body mass index, functional status, and mortality attrition.

**Figure 3:**
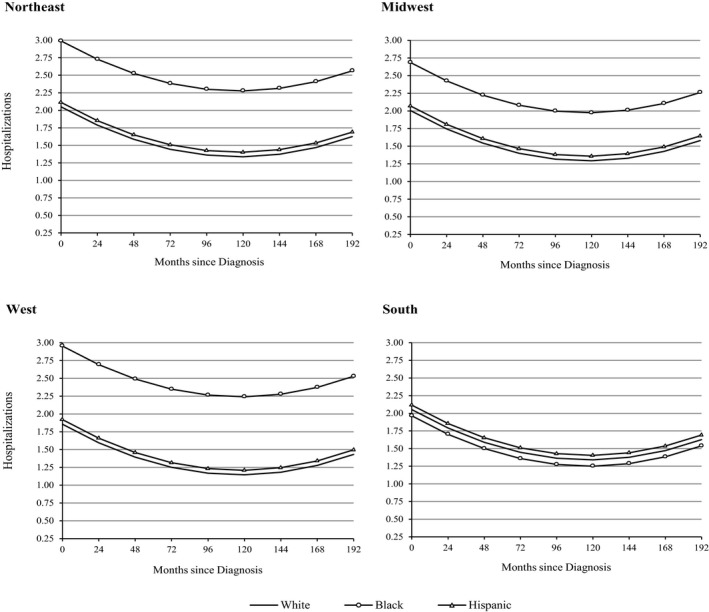
Predicted trajectories of hospitalization by race, ethnicity, and geographic region in US women with heart failure. Adjusted estimates account for foreign‐born status, urban residence, education, household income, health insurance, marital status, anxiety/emotional problems, smoking history, alcohol consumption, exercise, medication use, age at diagnosis, cardiovascular comorbidity (angina and acute myocardial infraction), other comorbidities (hypertension, diabetes mellitus, stroke, lung disease, and cancer), body mass index, functional status, and mortality attrition.

A final set of analyses found significant interactions for racial/ethnic differences in trajectories of hospitalization in men (urban‐rural residence) and women (geographic residence) following the diagnosis of HF (Table S5). In men, we found that blacks in urban areas (black×urban=0.78; 95% CI, 0.08–1.48 [*P*=0.030]) had significantly more hospitalizations than whites and Hispanics throughout most of their illness (Figure 2). In rural areas, however, white men were hospitalized significantly more than nonwhite men—at levels that were nearly identical to black men in urban areas. Patterns were similar in women but not significant. In women (Figure 3), we found that blacks were hospitalized significantly more than whites and Hispanics in most US regions (black×Northeast=1.03; 95% CI, 0.22–1.83 [*P*=0.012]; black×Midwest=0.77; 95% CI, 0.13–1.40 [*P*=0.017]; and black×West=1.19; 95% CI, 0.12–2.25 [*P*=0.028]). In southern states, however, there was no evidence of racial/ethnic differences in the number of hospitalizations in women with diagnosed HF. Patterns were similar in men but not significant, and no other significant interactions by race/ethnicity were found in men or women.

## Discussion

Racial/ethnic disparities in (re)hospitalizations among HF patients have been identified as actionable targets to reduce healthcare costs and improve patient outcomes.^3^, ^4^, ^44^, ^45^ Our study is the first prospective investigation of racial/ethnic differences in individual trajectories of hospitalization following the diagnosis of HF. We found that the number and pattern of hospitalizations over time was significantly different in white, black, and Hispanic men and women. The differences varied by place of residence and persisted after adjustment for multiple sociodemographic, behavioral, psychosocial, and physiological risk factors. The findings have important implications that may be crucial to planning the immediate and long‐term delivery of care in patients with HF to reduce potentially preventable hospitalizations.

We found that hospitalizations were elevated during the initial period after the diagnosis of HF, declined during the ensuing interval, and subsequently increased at the later stages of illness. This general pattern is consistent with the etiology of HF that may be diagnosed with the exacerbation of symptoms and/or incidence of another condition, such as acute myocardial infarction.^1^, ^46^, ^47^ The significant decline in hospitalizations after HF diagnosis in Hispanic men can be interpreted with mixed implications. On one hand, the precipitous drop in hospitalizations among Hispanic men suggests adequate outpatient management of their HF for lengthy periods of the disease. On the other hand, disproportionately low levels of hospital utilization may be indicative of obstacles to access in care and/or delays in receiving treatment among Hispanic men.^12^, ^48^ As a result, we suspect that the number of hospitalizations may rapidly escalate at later stages with the progression of HF (decompensation) and the advancement of untreated symptoms and/or related comorbidities. Therefore, we encourage future studies to assess whether more vigilant screening and follow‐up of Hispanic men after their diagnosis may prevent acute exacerbations of HF and/or other health conditions requiring hospitalization at later periods.

Our results for women showed that hospitalizations were significantly higher among blacks than among whites and Hispanics following the diagnosis of HF. Consistent with previous research, black women in our study received a diagnosis of HF at younger ages and were more likely to have comorbidities, limitations in activities of daily living, and a lack of health insurance compared with white women.^1^, ^7^, ^9^, ^17^, ^18^, ^49^ However, racial differences in hospitalization largely remained despite adjustments for these and other risk factors. It is also unclear whether differences in the access, utilization, and/or quality of care—eg, prescription/use of angiotensin‐converting enzyme inhibitors, tests of left ventricular ejection fraction, and receiving care from a cardiologist—may have played a role.^17^, ^18^

Our findings also demonstrated significant geographic variations that have implications for identifying locations for targeted interventions. In men, we found that blacks in urban areas had significantly more hospitalizations than whites and Hispanics throughout most of their illness. In rural areas, however, white men were hospitalized significantly more than nonwhite men—at levels that were nearly identical to black men in urban areas. The explanation for these patterns is not entirely clear; however, some research has shown that rural patients are hospitalized with less severe symptoms of HF than urban patients.^50^ Another possible explanation for these findings may be attributable to differences in access and/or utilization of ambulatory care in rural and urban areas.^18^, ^51^, ^52^, ^53^ However, more research is needed to further evaluate these or other contributing factors.

In women, we found that black‐white disparities in hospitalization were greatest in the West and Northeast regions of the United States and were not observed in Southern regions. These regional variations are not necessarily incongruent with recent research^5^, ^10^, ^16^, ^54^ and the reasons for the discrepancy are presumably 2‐fold. First, the HRS followed hospitalizations from a prospective cohort of adults and did not include hospitalizations for patients with newly incident HF. Second, the current study examined changes in individuals' number of hospitalizations over time and not aggregated rates of hospitalization. Prior analyses of Medicare claims and hospital discharge data have combined incident and existing cases of HF, and, in doing so, did not distinguish the number of hospitalizations that are shown to vary over time and the stage of illness. Based on our findings, higher overall rates of HF hospitalization in the South (at the population level) were not indicative of more hospitalizations over the course of the illness (at the individual level, shown here). Instead, we found that black‐white differences in the number of hospitalizations were most pronounced in the Northeast and West. It is not clear whether or to what extent these differences may be related to possible regional variations in the quality of HF care.^17^, ^55^ We encourage additional studies to further validate these findings of patient‐level changes in hospitalizations following an HF diagnosis.

### Study Strengths

A major strength of this study was the nationally representative panel data of older adults, which included frequent follow‐up over as many as 16 years. Much of the existing literature on HF (re)hospitalizations has been based almost exclusively on claims data and hospital discharge records.^4^, ^12^, ^13^, ^17^, ^19^ Although aggregated studies of Medicare beneficiaries and hospitalized patients have been optimal for examining readmission rates, these data are largely absent of information on patients outside of the hospital and have provided limited insights into the prospective patterning of HF hospitalizations during the course of illness. Our study was unique in providing new knowledge about changes in the number (and timing) of hospitalizations after the diagnosis of HF. Our study also was unique in estimating multilevel models that included time‐varying measures of nearly 2 dozen sociodemographic, psychosocial, behavioral, and physiological covariates. Previous studies have often relied on census data to approximate patients' background and social environment^7^, ^16^, ^54^, ^56^, ^57^ and few studies have included such a wide array of patient‐level characteristics.

### Study Limitations

Despite the strengths of this study, several limitations should be acknowledged. First, we recognize that the analyses are based on self‐reported number of hospitalizations. Although we cannot confirm these data, we believe inaccuracies were limited because of (1) the relatively short intervals between surveys, (2) the seriousness and infrequency of hospitalization (≈90% were hospitalized ≤3 times at each follow‐up), and (3) the HRS design that gathered detailed information on all healthcare utilization and expenditures for each patient. Preliminary analyses also showed that hospitalizations reported in the HRS were comparable with hospitalizations reported from national hospital discharge data, Medicare beneficiaries, and other US data (Tables S1 and S2). Nevertheless, we acknowledge that patients' reports of inpatient hospitalizations are less precise than administrative data. Relatedly, we recognize that the analyses are based on self‐reported diagnoses of HF that were not formally adjudicated, and that we lacked clinical data on HF subtype (HF with a preserved ejection fraction versus HF with reduced ejection fraction), severity (ie, New York Heart Association class), and pathogenesis (ie, ischemic versus nonischemic).

We also acknowledge that differential mortality in patients with HF is an important factor in trajectories of hospitalization. A notable advantage of our mixed models was the inclusion—and identification—of patients who died and thereby alleviated potential bias caused by the exclusion of cases attributable to mortality. In addition, as previously shown, we included an indicator for mortality in the estimation of the level 2 models to control for differences in trajectories between patients who survived and died. It also controlled for these differences on the effects of other covariates in the models.^40^, ^41^, ^42^ Preliminary analyses also accounted for differential mortality according to race and changes over time—including interaction terms—however, these indicators were not significant and did not alter the findings.

Finally, although the study included a wide range of covariates, it is possible that additional unmeasured factors may have contributed to the findings. For example, data were not available for the treatment and control of hypertension, diabetes mellitus, and hyperlipidemia, or measures of other therapeutic interventions (eg, revascularization and cardiac resynchronization) that may influence the number of hospitalizations. We also lacked data on hospital characteristics, causes of admissions, length of hospitalization, specific HF therapies, access to primary/specialty care, and other measures related to the quality of care. Therefore, we encourage additional studies to further examine the factors that may be contributing to racial/ethnic differences in hospitalizations.

## Conclusions

High rates of potentially preventable hospitalizations in adults with HF have put enormous strain on the US healthcare system. We found significant racial/ethnic differences in trajectories of hospitalization following the diagnosis of HF in US men and women. The disparities varied by geography and the differences persisted after adjustment for multiple risk factors. Results from our study provide valuable new evidence to help clinicians target patients with HF who may be at especially high risk of hospitalization during the course of treatment. An important area for future research will be to investigate the mechanisms underlying the findings in this study and to identify possible interventions to reduce these risks.

## Author Contributions

Dupre and Xu had full access to the data in the study and take responsibility for the accuracy of the data analysis. Study concept and design: Dupre. Acquisition of data: Xu. Analysis and interpretation of data: Dupre, Gu, and Xu. Drafting of the article: Dupre, Xu, Willis, Curtis, and Peterson. Critical revision of the article for important intellectual content: Dupre, Gu, Willis, Curtis, and Peterson. Statistical analysis: Dupre and Xu. Administrative, technical, or material support: Dupre and Willis. The views expressed in this article are those of the authors and do not necessarily reflect those of Duke University or the United Nations.

## Sources of Funding

Support for this study was provided by the National Heart, Lung, and Blood Institute grant K01HL114750. The National Heart, Lung, and Blood Institute had no role in the design and conduct of the study; collection, management, analysis, and interpretation of the data; preparation, review, or approval of the article; or decision to submit the article for publication.

## Disclosures

None.

## Supporting information


**Figure S1.** Comparisons of heart failure (HF) prevalence by age and sex in National Health and Nutrition Examination Survey (NHANES) and HRS (Health and Retirement Study) data sets in selected years.
**Figure S2.** Study participants from the HRS (Health and Retirement Study).
**Figure S3.** Survival probabilities by race and ethnicity in US men and women with heart failure (HF).
**Table S1.** Hospitalization Rates (Per 1000 Population) by Age Group in the HRS and Other Documented Sources in Selected Years
**Table S2.** Number of Hospitalizations in the Past Year in the HRS and NHIS in Selected Years
**Table S3.** Parameter Estimates for Trajectories of Hospitalization Over Time in US Older Adults With HF
**Table S4.** Parameter Estimates for Racial/Ethnic Differences in Trajectories of Hospitalization Over Time in US Men and Women With HF
**Table S5.** Parameter Estimates for Racial/Ethnic Differences in Trajectories of Hospitalization in US Men and Women With HF by Place of Residence
