# Supplementary material for: Racial and Ethnic Differences in Trajectories of Hospitalization in US Men and Women With Heart Failure
Source: J Am Heart Assoc. 2017 Nov 16;6(11):e006290. doi: 10.1161/JAHA.117.006290 (PMC5721744; doi:10.1161/JAHA.117.006290)
Supplement: Supplementary file 1 — Figure S1. Comparisons of heart failure (HF) prevalence by age and sex in National Health and Nutrition Examination Survey (NHANES) and HRS (Health and Retirement Study) data sets in selected years. Figure S2. Study participants from the HRS (Health and Retirement Study). Figure S3. Survival probabilities by race and ethnicity in US men and women with heart failure (HF). Table S1. Hospitalization Rates (Per 1000 Population) by Age Group in the HRS and Other Documented Sources in Selected Years Table S2. Number of Hospitalizations in the Past Year in the HRS and NHIS in Selected Years Table S3. Parameter Estimates for Trajectories of Hospitalization Over Time in US Older Adults With HF Table S4. Parameter Estimates for Racial/Ethnic Differences in Trajectories of Hospitalization Over Time in US Men and Women With HF Table S5. Parameter Estimates for Racial/Ethnic Differences in Trajectories of Hospitalization in US Men and Women With HF by Place of Residence [file JAH3-6-e006290-s001.pdf]

SUPPLEMENTAL MATERIAL

Figure S1. Comparisons of Heart Failure Prevalence by Age and Sex in NHANES and HRS Datasets in Selected Years

A.

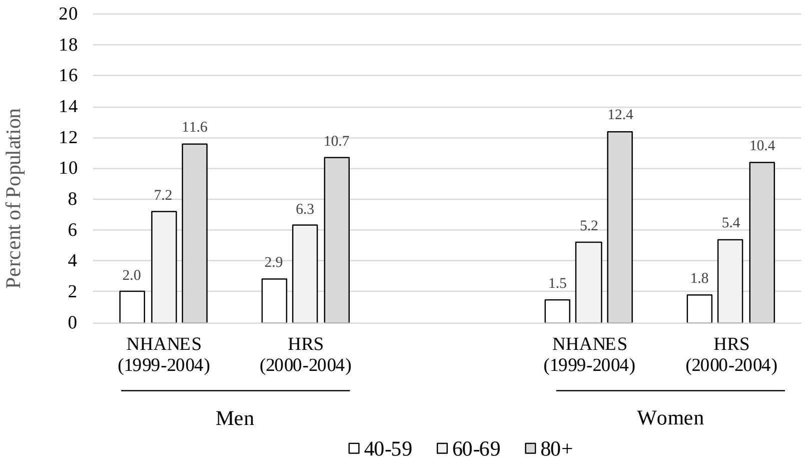

B.

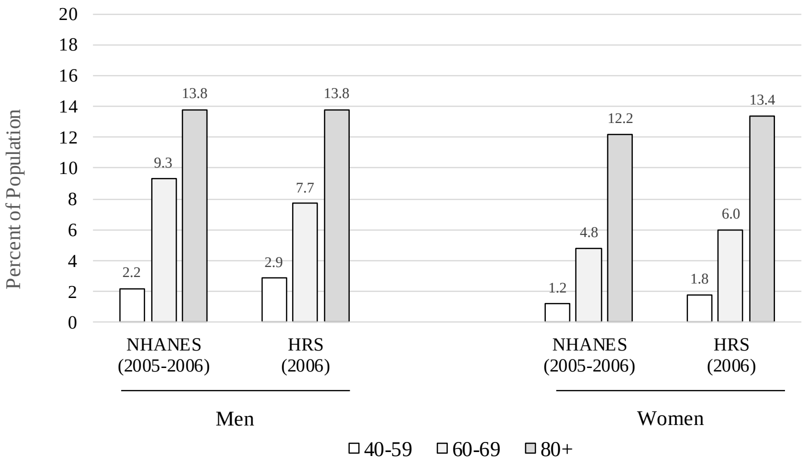

Source (A): NHANES data derived from: Rosamond, W. et al; American Heart Association Statistics Committee and Stroke Statistics Subcommittee. Circulation. 2007;115:e69-e171 (page e121; Chart 7-1). HRS data derived from average weighted prevalence in survey years 2000, 2002, and 2004.

Source (B): NHANES data derived from: Lloyd-Jones, D. et al; American Heart Association Statistics Committee and Stroke Statistics Subcommittee. Circulation. 2009;119:e21-e181 (page e103; Chart 8-1). HRS data derived from weighted prevalence in survey year 2006.

Figure S2. Study Participants from the Health and Retirement Study

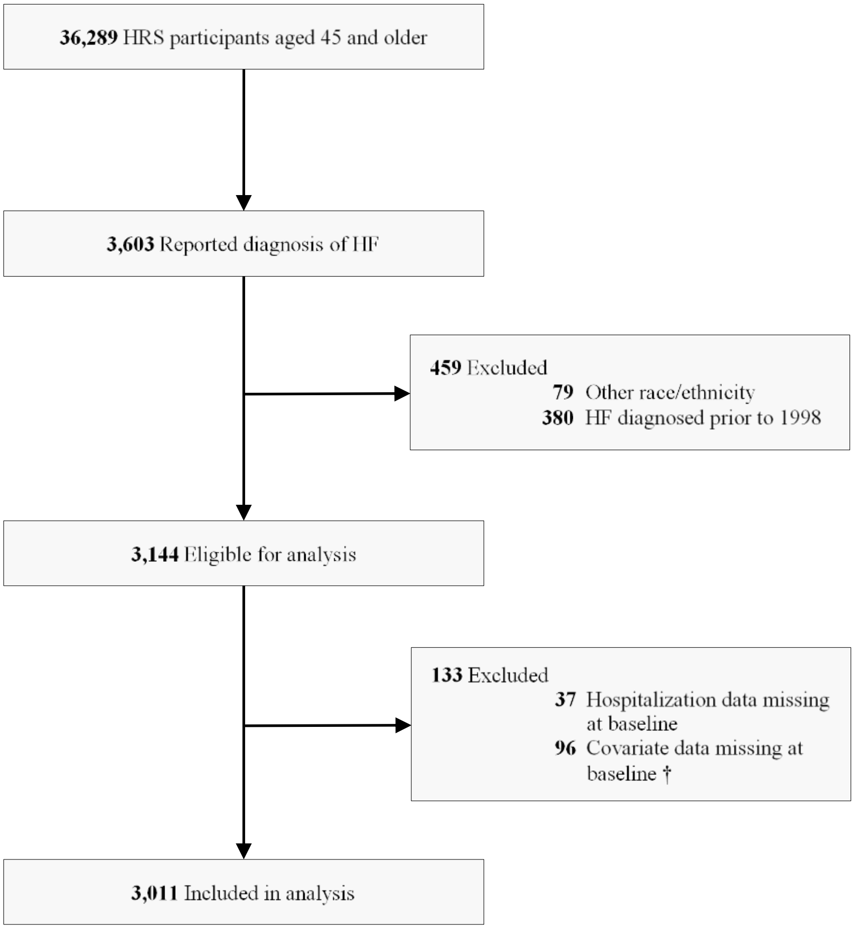

Abbreviations: HRS, Health and Retirement Study; HF, heart failure.
† Values for missing data are not mutually exclusive and included body mass index (n=28), smoking status (n=23), chronic illnesses (n=11), geographic region (n=10), drinking status (n=7), marital status (n=6), medication use (n=6), urban residence (n=5), foreign-born status (n=4), exercise (n=3), health insurance (n=1), and anxiety/emotional problems (n=1).

Figure S3. Survival Probabilities by Race and Ethnicity in U.S. Men and Women with Heart Failure

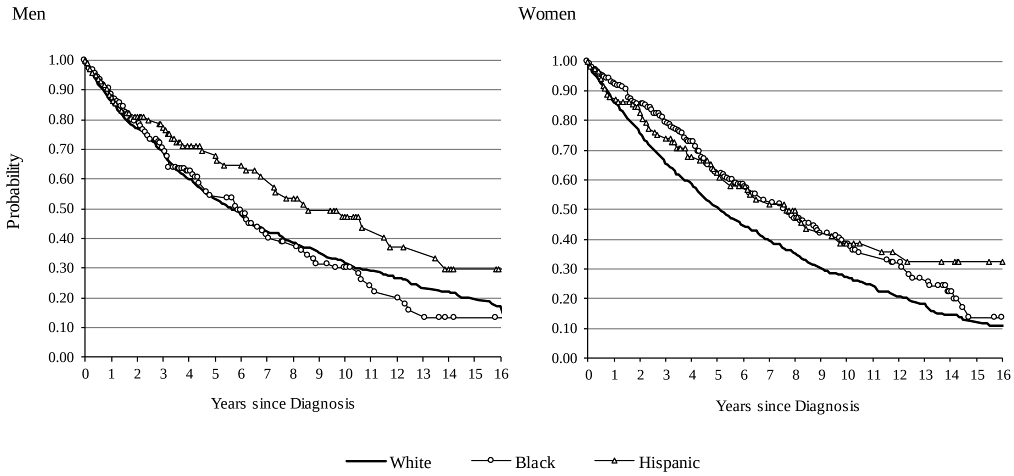

Note: Differences are statistically significant for men (p = .038) and women (p < .001) based on log-rank tests. Overall survival was 49.2% in men and 48.3% in women after 5 years.

Table S1. Hospitalization Rates (per 1,000 population) by Age Group in the HRS and Other Documented Sources in Selected Years

	HRS Data				
	All Participants 2000-2006	Medicare FFS Beneficiaries 2000-2006	Hospital Discharge Data * 2000	Hospital Discharge Data * 2006	Medicare FFS Beneficiaries # 2000-2006
**Age Group**					
Age 65-74	282.4	294.8	254.6	253.4	264.0
Age 75-84	398.1	402.2	412.4	402.6	392.5
Age 85+	515.0	529.7	605.1	571.1	580.1

Abbreviations: HRS, Health and Retirement Study; FFS, Fee-for-Service.
Note: Estimates for HRS data were based on the assumptions that hospitalizations of participants in each age group were stable within the 2-year study intervals and that hospitalizations among deceased in the interval were the same as those in the 1-2 years prior to death.
*Source: Buie VC, Owings MF, Defrances CJ, Golosinskiy A. National hospital discharge survey: 2006 annual summary. Vital Health Stat 13. 2010;(168):1-79. https://www.cdc.gov/nchs/data/series/sr_13/sr13_168.pdf
#Source: Gorina Y, Pratt LA, Kramarow EA, Elgaddal N. Hospitalization, Readmission, and Death Experience of Noninstitutionalized Medicare Fee-for-service Beneficiaries Aged 65 and Over. Natl Health Stat Report. 2015;(84):1-24. https://www.cdc.gov/nchs/data/nhsr/nhsr084.pdf

Table S2. Number of Hospitalizations in the Past Year in the HRS and NHIS in Selected Years

	2000		2006		2012	
	HRS	NHIS *	HRS	NHIS #	HRS	NHIS ^
**Number of Hospitalizations**						
None	82.9	81.9	81.5	82.9	82.4	84.2
1	12.7	12.3	13.3	11.8	12.6	10.5
2	3.2	3.4	3.8	3.0	3.5	3.2
3+	1.1	2.3	1.3	2.2	1.4	2.2

Abbreviations: HRS, Health and Retirement Study; NHIS, National Health Interview Survey.
Note: Values reported as percentages. Rates for HRS were estimated in a given year based on an equal distribution of hospitalizations in the 2-year interval.
*Source: Schoenborn CA, Adams PF, Schiller JS. Summary health statistics for the U.S. population: National Health Interview Survey, 2000. Vital Health Stat 10. 2003;(214):1-83. https://www.cdc.gov/nchs/data/series/sr_10/sr10_214.pdf
#Source: Adams PF, Lucas JW, Barnes PM. Summary health statistics for the U.S. population: National Health Interview Survey, 2006. Vital Health Stat 10. 2008;(236):1-104. https://www.cdc.gov/nchs/data/series/sr_10/sr10_236.pdf
^Source: Adams PF, Kirzinger WK, Martinez M. Summary health statistics for the U.S. population: National Health Interview Survey, 2012. Vital Health Stat 10. 2013;(259):1-95. https://www.cdc.gov/nchs/data/series/sr_10/sr10_259.pdf

Table S3. Parameter Estimates for Trajectories of Hospitalization Over Time in U.S. Older Adults with Heart Failure

	Model 1	Model 2	Model 3	Model 4
**Fixed-Effects Parameters**				
Intercept	1.80*** (1.71 to 1.88)	2.08*** (1.96 to 2.20)	2.36*** (2.19 to 2.53)	2.21*** (1.99 to 2.44)
Time		-0.12*** (-0.15 to -0.08)	-0.35*** (-0.45 to -0.25)	-0.22** (-0.36 to -0.09)
$Time^2$			0.03*** (0.02 to 0.05)	0.02* (0.00 to 0.04)
Male				0.32 (-0.01 to 0.66)
Male x Time				-0.27** (-0.47 to -0.07)
Male x $Time^2$				0.03* (0.00 to 0.06)
**Random-Effects Variance Components**				
Level 1: Within-person	5.10*** (4.90 to 5.31)	5.10*** (4.90 to 5.30)	5.09*** (4.90 to 5.30)	5.09*** (4.89 to 5.30)
Level 2: Intercept	4.80*** (4.18 to 5.53)	5.40*** (4.74 to 6.15)	5.14*** (4.48 to 5.89)	5.17*** (4.51 to 5.92)
Level 2: Slope	0.03*** (0.01 to 0.09)	0.08*** (0.05 to 0.13)	0.07*** (0.04 to 0.12)	0.07*** (0.04 to 0.12)
**Goodness-of-Fit**				
BIC value	42583.38	42549.23	42534.91	42552.69

Abbreviations: BIC, Bayesian information criterion.
Note: Estimated coefficients (95% confidence intervals) are reported. Time and $Time^2$ indicate linear and quadratic slope parameters, respectively.
* $p < 0.05$, ** $p < 0.01$, *** $p < 0.001$

Table S4. Parameter Estimates for Racial/Ethnic Differences in Trajectories of Hospitalization Over Time in U.S. Men and Women with Heart Failure

	Men		Women	
	Model 1	Model 2	Model 1	Model 2
**Fixed-Effects Parameters**				
Intercept	2.46*** (2.19 to 2.73)	3.71*** (2.27 to 5.15)	2.18*** (1.94 to 2.41)	2.87*** (1.43 to 4.31)
Time	-0.43*** (-0.58 to -0.28)	-0.59*** (-0.74 to -0.44)	-0.25*** (-0.39 to -0.11)	-0.35*** (-0.49 to -0.20)
Time^2	0.03*** (0.01 to 0.05)	0.05*** (0.03 to 0.07)	0.03** (0.01 to 0.05)	0.03** (0.01 to 0.05)
Black	0.21 (-0.12 to 0.55)	0.10 (-0.21 to 0.41)	0.28* (0.00 to 0.55)	0.29* (0.01 to 0.57)
Hispanic	0.57 (-0.33 to 1.46)	0.42 (-0.49 to 1.34)	-0.09 (-0.51 to 0.33)	0.06 (-0.40 to 0.52)
Hispanic x Time	-0.52* (-0.99 to -0.05)	-0.51* (-0.99 to -0.03)		
Hispanic x Time^2	0.06* (0.00 to 0.12)	0.06* (0.00 to 0.13)		
**Random-Effects Variance Components**				
Level 1: Within-person	4.41*** (4.16 to 4.67)	4.24*** (4.00 to 4.49)	5.55*** (5.27 to 5.84)	5.55*** (5.26 to 5.85)
Level 2: Intercept	8.20*** (7.05 to 9.53)	7.88*** (6.77 to 9.17)	3.27*** (2.67 to 4.00)	2.96*** (2.37 to 3.69)
Level 2: Slope	0.24*** (0.17 to 0.35)	0.32*** (0.24 to 0.44)	0.00*** (0.00 to 0.06)	0.01*** (0.00 to 0.04)
**Goodness-of-Fit**				
BIC value	42583.38	42549.23	42534.91	42552.69

Abbreviations: BIC, Bayesian information criterion.
Note: Estimated coefficients (95% confidence intervals) are reported. Time and Time^2 indicate linear and quadratic slope parameters, respectively.
Model 1 is unadjusted. Model 2 adjusts for foreign-born status, geographic region, urban residence, education, household income, health insurance, marital status, anxiety/emotional problems, smoking history, alcohol consumption, exercise, medication use, age at diagnosis, cardiovascular comorbidity (angina and acute MI), other comorbidities (hypertension, diabetes, stroke, lung disease, and cancer), body mass index, functional status, and mortality attrition.
* $p < 0.05$, ** $p < 0.01$, *** $p < 0.001$

Table S5. Parameter Estimates for Racial/Ethnic Differences in Trajectories of Hospitalization in U.S. Men and Women with Heart Failure by Place of Residence

	Men		Women	
**Fixed-Effects Parameters**				
Intercept	3.60***	(2.17 to 5.04)	2.83***	(1.38 to 4.27)
Time	-0.59***	(-0.74 to -0.44)	-0.35***	(-0.50 to -0.21)
$Time^2$	0.05***	(0.03 to 0.07)	0.03**	(0.01 to 0.05)
Black	-0.51	(-1.14 to 0.12)	-0.09	(-0.44 to 0.26)
Hispanic	0.42	(-0.49 to 1.34)	0.06	(-0.41 to 0.54)
Hispanic x Time	-0.51*	(-1.00 to -0.03)		
Hispanic x $Time^2$	0.06*	(0.00 to 0.13)		
Urban (vs. rural)	-0.30**	(-0.52 to -0.08)	0.01	(-0.23 to 0.24)
Urban x Black	0.78*	(0.08 to 1.48)		
Geographic region				
Northeast (vs. South)	0.05	(-0.24 to 0.34)	-0.00	(-0.37 to 0.36)
Midwest (vs. South)	0.16	(-0.07 to 0.40)	-0.05	(-0.33 to 0.24)
West (vs. South)	0.17	(-0.10 to 0.44)	-0.20	(-0.54 to 0.15)
Northeast x Black			1.03*	(0.22 to 1.83)
Midwest x Black			0.77*	(0.13 to 1.40)
West x Black			1.19*	(0.12 to 2.25)
**Random-Effects Variance Components**				
Level 1: Within-person	4.24***	(4.00 to 4.49)	5.54***	(5.25 to 5.84)
Level 2: Intercept	7.89***	(6.77 to 9.18)	2.95***	(2.37 to 3.68)
Level 2: Slope	0.33***	(0.24 to 0.44)	0.01***	(0.00 to 0.04)
**Goodness-of-Fit**				
BIC value	18746.81		23177.05	

Abbreviations: BIC, Bayesian information criterion.
Note: Estimated coefficients (95% confidence intervals) are reported. Time and $Time^2$ indicate linear and quadratic slope parameters, respectively.
Models also adjusted for foreign-born status, education, household income, health insurance, marital status, anxiety/emotional problems, smoking history, alcohol consumption, exercise, medication use, age at diagnosis, cardiovascular comorbidity (angina and acute MI), other comorbidities (hypertension, diabetes, stroke, lung disease, and cancer), body mass index, functional status, and mortality attrition.
* $p < 0.05$, ** $p < 0.01$, *** $p < 0.001$
